# Predicting the Tool Wear of a Drilling Process Using Novel Machine Learning XGBoost-SDA

**DOI:** 10.3390/ma13214952

**Published:** 2020-11-04

**Authors:** Mahdi S. Alajmi, Abdullah M. Almeshal

**Affiliations:** 1 Department of Manufacturing Engineering Technology, College of Technological Studies, Public Authority for Applied Education and Training, Safat 13092, Kuwait; 2 Department of Electronic Engineering Technology, College of Technological Studies, Public Authority for Applied Education and Training, Safat 13092, Kuwait; am.almeshal@paaet.edu.kw

**Keywords:** machine learning, flank wear prediction, XGBoost, SDA, optimization, machining parameters, drilling process, support vector machines, artificial neural networks

## Abstract

Tool wear negatively impacts the quality of workpieces produced by the drilling process. Accurate prediction of tool wear enables the operator to maintain the machine at the required level of performance. This research presents a novel hybrid machine learning approach for predicting the tool wear in a drilling process. The proposed approach is based on optimizing the extreme gradient boosting algorithm’s hyperparameters by a spiral dynamic optimization algorithm (XGBoost-SDA). Simulations were carried out on copper and cast-iron datasets with a high degree of accuracy. Further comparative analyses were performed with support vector machines (SVM) and multilayer perceptron artificial neural networks (MLP-ANN), where XGBoost-SDA showed superior performance with regard to the method. Simulations revealed that XGBoost-SDA results in the accurate prediction of flank wear in the drilling process with mean absolute error (MAE) = 4.67%, MAE = 5.32%, and coefficient of determination R^2^ = 0.9973 for the copper workpiece. Similarly, for the cast iron workpiece, XGBoost-SDA resulted in surface roughness predictions with MAE = 5.25%, root mean square error (RMSE) = 6.49%, and R^2^ = 0.975, which closely agree with the measured values. Performance comparisons between SVM, MLP-ANN, and XGBoost-SDA show that XGBoost-SDA is an effective method that can ensure high predictive accuracy about flank wear values in a drilling process.

## 1. Introduction

Production companies are attempting to boost product quality as well as to reduce operating costs. Online real-time control and monitoring of drilling processes was proposed as an effective method to minimize manufacturing costs [1]. The drilling process is one of the most used processes in manufacturing across various industries such as automotive and aerospace sectors [2]. The drilling process is produced by drilling and boring of material removal and is related to conventional drill bits geometry. One of the common problems across processes such as drilling, milling, and turning is the tool wear [3,4]. The worn tools lower the production quality and result in drilling holes and may lead to damage to both the workpiece and the machine. In addition, it may result in increasing the cutting force that results in raising the temperature and accelerate the tool wear [5,6].

There exist various types of drill tool wear such as flank wear, chisel edge wear, margin and crater wear [7]. Numerous investigations of tool wear in drilling processes exists in literature. Wang et al. [8] investigated the wear on three different drills (uncoated, diamond coated and AlTiN coated carbide) used in the drilling of carbon fiber reinforced composites (CFRP). Imran et al. [9] studied the impact of the tool wear on the surface integrity in a micro-drilling wet and dry cutting processes. Similarly, Xiang et al. [10] presented a finite element simulation of the drilling tool wear of SiCp/Al6063 composites.

The prediction of tool wear is an essential tool to monitor the process and control the quality in manufacturing process [11]. With the aid of machine learning, soft computational approach, and the existing experimental datasets, it is possible to utilize the tool wear of various processes to a high extent of accuracy. Recently, artificial neural networks (ANN) have been extensively utilized and proven to be an effective tool to predict the tool wear based when trained on an experimental dataset. Due to its proven efficiency and self-learning capabilities, it has been described in literature as a promising quick solution for tool wear prediction. Zhao et al. [12] have presented an ANN for predicting the optimal rate of penetration (RoP). The results have shown that ANN can be utilized to provide an accurate prediction of the optimal RoP parameters in drilling process. Kong et al. [13] have presented a robust machine learning prediction approach to predict the flank wear on various cutting process conditions. The authors have utilized kernel principal component analysis with an integrated radial basis function (KPCA-IRBF) and Gaussian regression to obtain online accurate tool wear parameters.

Similarly, Chen et at [14] have proposed a deep belief network (DBN) for predicting a cutting tool flank wear. The proposed method was compared with state-of-art machine learning approaches such as support vector regression (SVR) and ANN and was shown to provide superior performance in terms of statistical metrics such as mean square error (MSE) and coefficient of determination (R^2^).

Machine learning approaches and Evolutionary optimization algorithms were applied to predict parameters in material sciences such as flank wear [15,16]. Yang et al. [16] have proposed a co-evolutionary particle swarm optimization-based selective network ensemble (E-CPSOSEN) to predict the flank wear in drilling operations. Several simulations were carried out and the results were compared with ANN prediction method. The E-CPSOSEN have shown a better accuracy than the ANN approach in terms of performance metrics. Similarly, adaptive particle swarm optimization (APSO) to predict the tool wear in drilling process was introduced by Chen et al. [17]. The APSO was integrated with least square support vector machine (LS-SVM) and have resulted in a better prediction accuracy than the LS-SVM approach.

Adaptive neuro-fuzzy inference systems with genetic algorithm (ANFIS-GA) was utilized by Saw et al. [18] for prediction of optimal tool wear in a drilling process. The ANFIS-GA was shown to provide fast and accurate results in comparison with evolutionary optimization methods such as GA. Nature inspired optimization techniques were also introduced as a tool to predict the tool wear such as the DNA-based computing (DBC) that was presented by Addona et al. [19]. Patra et al. [20] have presented an ANN for tool wear prediction in a peck drilling process and have compared the results with the experimental datasets. The achieved predictions closely match the experimental dataset and that validated the feasibility of the proposed approach.

Alajmi and Almeshal [21] have utilized a novel quantum particle swarm optimization of an ANFIS model (ANFIS-QPSO) to predict the surface roughness values of the dry and cryogenic turning process parameters. The proposed approach combines the strengths of the ANN in self-learning and the fast convergence of QPSO in obtaining optimal parameters to provide highly accurate prediction results. With all the aforementioned studies, it can be noted that the machine learning approaches and specifically ANN based prediction models are promising techniques that provide robust, highly accurate, and fast prediction of various process parameters [22,23,24,25,26,27].

In this research, we propose a novel spiral dynamic optimized extreme gradient boosting machine learning algorithm (XGBoost-SDA) for predicting the tool wear in the drilling of copper and cast iron workpieces. There is a gap in the literature on using XGBoost to predict flank wear in the drilling process to the best of our knowledge. Additionally, no previous study has investigated the use of the XGBoost-SDA approach for this purpose. The present study examines the accuracy of XGBoost-SDA predictions of the experimental dataset of two workpieces of copper and cast iron compared with state-of-the-art prediction algorithms such as the support vector machine (SVM) and multilayer perceptron ANN (MLP-ANN). In the next section, the methodology of the proposed XGBoost-SDA approach is outlined with the nomenclature presented in Table 1. The simulation results present the predicted results and highlight the prediction accuracy of XGBoost-SDA when compared with the SVM and MLP-ANN approaches.

## 2. Methodology

### 2.1. Extreme Gradient Boosting (XGBoost) Algorithm

XGBoost is a supervised machine learning algorithm developed by [28], which has caught the interest of researchers in various fields [29,30,31,32] due to its performance in terms of speed and accuracy. The algorithm has yielded state-of-the-art results on many benchmark problems due to its scalability, speed, distributed computing features and its ability to handle sparse data. XGBoost is an ensemble algorithm that aggregates weak learners, classification, and regression trees (CART), to build a powerful meta-learner for boosting performance. Let $D=\left\{\left(x_{i},y_{i}\right)\right\}$ define a dataset with *n* samples and *m* features $\left|D\right|=n,\;x_{i}\epsilon R^{m},\;y_{i}\epsilon R$; the XGBoost algorithm ensembles *K* additive functions to predict the outputs as:

(1) $${\hat{y}}_{i}=\phi \left(x_{i}\right)=\overset{K}{\underset{k=1}{\sum }}f_{k}\left(x_{i}\right)\;f_{k}\;\epsilon \;F$$

in which the space of regression trees is denoted by F as:

(2) $$F=\left\{\;f\left(x\right)=\omega_{q\left(x\right)}\right\}\;q:R^{m}\to T,\;\omega \in R^{T}$$

with q as the tree structure, T  and ω representing the number of leaf nodes and associated weights respectively. To minimize the prediction error, we defined the regularized objective function as:

(3) $$L\left(\phi \right)=\underset{i}{\sum }l\left({\hat{y}}_{i},y_{i}\right)+\underset{k}{\sum }\Omega \left(f_{k}\right)$$

with l as a differentiable convex loss function that defines the error between the actual and predicted values whereas Ω presents the penalization function defined as:

(4) $$\Omega \left(f_{k}\right)=\gamma T+\frac{1}{2}\lambda {\left|\left|w\right|\right|}^{2}$$

The selection of hyperparameters of the XGBoost greatly impacts the model’s predictive accuracy. Table 2 presents the hyperparameters of the XGBoost algorithm. Finding the optimal balance between these parameters by trial and error could be challenging. To overcome this challenge, we propose a novel XGBoost-SDA model to predict the tool wear of a drilling process.

In the next section, the spiral dynamic optimization algorithm is introduced and the integration with XGBoost to optimize the hyperparameters is presented.

### 2.2. Spiral Dynamics Optimisation Algorithm (SDA)

A spiral dynamic algorithm (SDA) is a metaheuristic algorithm, which was developed by Tamura and Yasuda [33] and is inspired by the spiral phenomena in nature. SDA was proven to outperform many metaheuristic search algorithms in its convergence speed and accuracy, due to its diversification and intensification search approach. In diversification it searches for good solutions within the search space while intensification is used to search for the optimal values around the best solutions. Table 3 presents the nomenclature of the SDA parameters. Algorithm 1 presents the SDA steps to obtain the optimal solution. 

Assuming *R* is a rotation matrix for the *n*-dimension SDA algorithm where it is defined as

(5) $$R^{n}{}_{i,j}(\theta_{i,j})=\begin{array}{cc} & i\;\;\;\;\;\;\;\;\;\;\;\;\;\;\;\;\;\;\;\;\;\;\;\;j\\ \begin{array}{l} i\\ j \end{array} & \left[\begin{array}{ccccccccccc} 1 & & & & & & & & & & \\ & \ddots & & & & & & & & & \\ & & 1 & & & & & & & & \\ & & & \cos\theta_{i,j} & & \cdots & & -\sin\theta_{i,j} & & & \\ & & & & 1 & & & & & & \\ & & & \vdots & & \ddots & & \vdots & & & \\ & & & & & & 1 & & & & \\ & & & \sin\theta_{i,j} & & \cdots & & \cos\theta_{i,j} & & & \\ & & & & & & & & 1 & & \\ & & & & & & & & & \ddots & \\ & & & & & & & & & & 1 \end{array}\right] \end{array}$$

The *n*-dimension spiral dynamic model is expressed using the rotational matrix as:

(6) $$x_{i}(k+1)=S_{n}(r_{},\theta )x_{i}(k)-(S_{n}(r,\theta )-I_{n})x^{*}$$

where

(7) $$S_{n}(r_{},\theta )x(k)=\prod_{i=1}^{n-1}(\prod_{j=1}^{i}R_{n-i,n+1-j}^{n}(\theta_{n-i,n+1-j}))$$

The *n*-dimension SDA optimization algorithm is then written as
**Algorithm 1:** Spiral dynamics optimization algorithm **Step 0:** PreparationSelect the number of search points m≥2
0≤θ<2π ,  0<r<1 of $S_{n}\left(r,\theta \right)$ and maximum number of iterations $k_{max}$
**Step 1:** InitializationSet initial points $x_{i}\left(0\right)\;\epsilon \;R^{n},\;i=1,2,\ldots ,m\;$ in the feasible region randomly and centered $x^{*}$
with $x^{*}=x_{i_{g}}\left(0\right)\;\;\;with\;\;\;i_{g}=\arg\underset{i}{\min}f\left(x_{i}\left(0\right)\right),\;i=1,2,\ldots ,m$
**Step 2:** Update x
$x_{i}\left(k+1\right)=S_{n}\left(r,\theta \right)x_{i}\left(k\right)-\left(S_{n}\left(r,\theta \right)-I_{n}\right)x^{*}$ for i=1,2,…,m
**Step 3:** Update $x^{*}$
$x^{*}=x_{i}{}_{g}\left(k+1\right)$ with $i_{g}=\arg\underset{i}{\min}f\left(x_{i}\left(0\right)\right),\;i=1,2,\ldots ,m$
**Step 4:** Check for the termination criteriaIf $k=\;k_{max}$ then terminate; otherwise set k=k+1 and return to Step 2.

**Figure 1:**
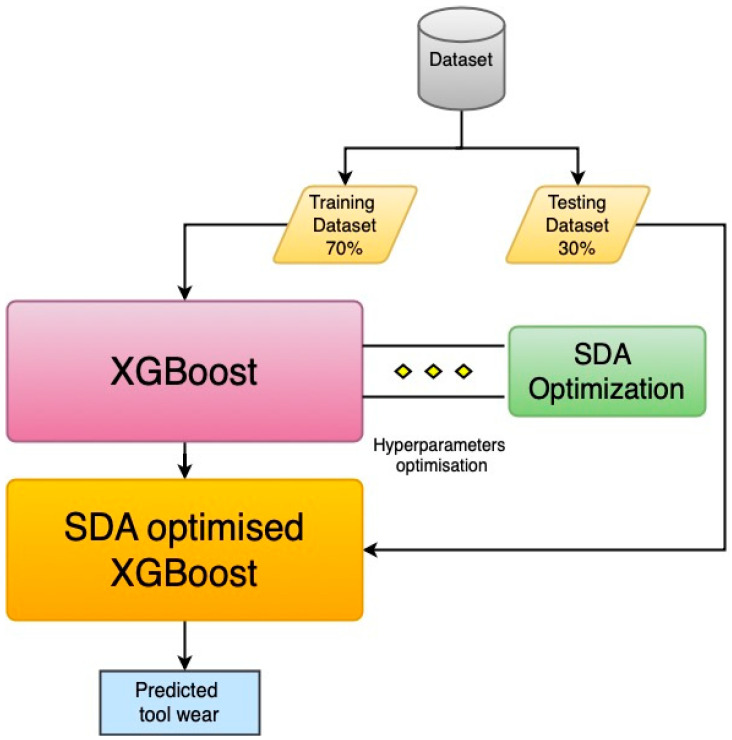
The flowchart of extreme gradient boosting with spiral dynamics optimization algorithm (XGBoost-SDA).

In this research, we propose a novel hybrid XGBoost-SDA algorithm for predicting the tool wear. Figure 1 illustrates the proposed XGBoost-SDA flowchart.

The process starts by splitting the dataset into training and testing datasets. The training dataset presents 70% of the data to train the algorithm, while the remaining 30% are used for testing and validating the algorithm for the prediction performance and accuracy. An initial XGBoost algorithm is trained by the training data and the hyperparameters and the prediction accuracy is evaluated by calculating the root mean square error (RMSE). The hyperparameters are then fed into the SDA algorithm to find the best hyperparameter values with those that correspond to the lowest RMSE value. The optimal hyperparameters are then used to initialize a new XGBoost algorithm, referred to as XGBoost-SDA, to predict the tool wear values of the testing data set with the lowest RMSE.

## 3. Results and Discussion

In this section, the analyzed performance of the XGBoost -SDA algorithm enabled tool wear prediction model for drill wear prediction is reported. Table 4, Table 5 and Table 6 present the experimental dataset, performance metrics of XGBoost-SDA and the prediction results of the flank wear for a copper workpiece respectively with 49 experimental trials. While Table 7, Table 8 and Table 9 present the experimental dataset, performance metrics of the XGBoost-SDA and the flank wear prediction results of a cast iron workpiece respectively with 63 experimental trials. 

**Figure 2:**
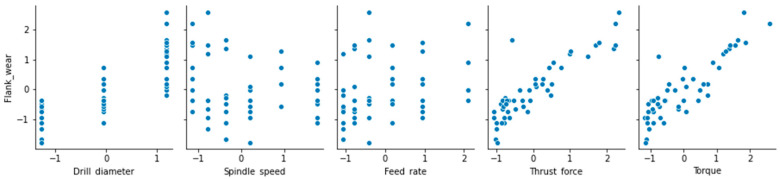
Pairwise relationship of different inputs with the flank wear of the copper workpiece.

The various trials provides combination of input parameters of spindle speed, drill diameter, feed rate, thrust force, and torque. Figure 2 presents the pair-wise relationship between the various inputs and the flank wear of the drilling process. It can be observed that the thrust force and torque have a linear relationship with the flank wear output variable; whereas the drill diameter, feed rate and spindle speed are in a non-linear relationship with the flank wear output variable.

High speed steel drill bits with different diameters (5, 7.5, and 10 mm) were used for drilling holes in the mild steel and copper workpieces. The spindle speed was incremented in six equally spaced intervals from 315 to 1000 rpm. Similarly, the feed rate was also varied in six steps from 0.13 to 0.71 mm/rpm. However, the type of wear in this machining process is adhesion due to its predominant wear factor in the drill cutting edges [34].

Different combinations of input parameters of spindle speed, feed rate and drill diameter were used to perform 49 trials of drilling process. The spindle speed values are within the range between 250–500 rpm and were varied in four steps. In addition, the feed rate was varied from 0.13 to 0.36 mm/rev in four steps. The drill diameter values were of 9, 10, 11, and 12 mm and were used to drill 15 mm thickness of cast iron workpiece. These three process parameters were used in 63 different combinations, and the corresponding output of the experimental setup noted in terms of thrust force, torque, and flank wear.

A comparison between the results of the proposed method (XGBoost-SDA) with SVM and MLP-ANN is provided in Table 5 and Table 8. The comparison highlights the statistical performance metrics of each method such as mean absolute error (MAE), root mean square error (RMSE) and the coefficient of determination (R^2^). In order to demonstrate the performance of XGBoost-SDA algorithm for tool wear prediction, two illustrative cases (copper and cast-iron workpiece), with datasets acquired from [17], were used in this simulation and its performance was compared with results and methods of the SVM and a MLP-ANN.

Table 6 and Table 9 present a comparison of the predicted values from the XGBoost-SDA, SVM, and MLP with the experimental values of the copper and cast iron workpieces, respectively. The results show that the predicted tool wear obtained by XGBoost-SDA closely matches the actual values of the measured tool wear compared to the SVM and MLP methods, which visually confirmed how well the XGBoost-SDA fitted the validation dataset.

However, as can be seen in Table 5 and Table 8 all of the algorithms performed well, with slight performance measures, in predicting the flank wear values of the drilling process. It should be noted that XGBoost-SDA showed considerably better predictive performance, which outperformed SVM, and MLP-ANN in terms of the three performance indicators. Moreover, in the case of the copper workpiece, to ensure the reliability and efficiency of the XGBoost-SDA compared to the SVM and MLP-ANN methods, it should be noted that the model resulted in a mean absolute error (MAE) of 4.67% that reflected the efficacy of the XGBoost-SDA model to predict the flank wear values to a credible extent. In addition, the RMSE (Root Mean Square Error) value was 5.32% and the coefficient of determination R^2^ was 0.9973 for the copper workpiece. For the cast iron workpiece, the results were 5.25% for RMS, 6.49% for MAE, and 0.9756 for R^2^, which reflects the good fit of the predicted values against the measured flank wear values.

**Figure 3:**
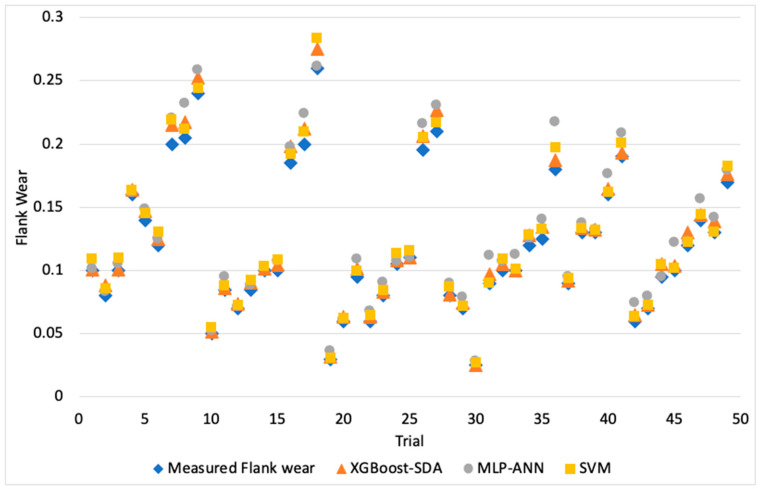
Flank wear values vs. predicted flank wear values of a drilling process (Copper workpiece).

**Figure 4:**
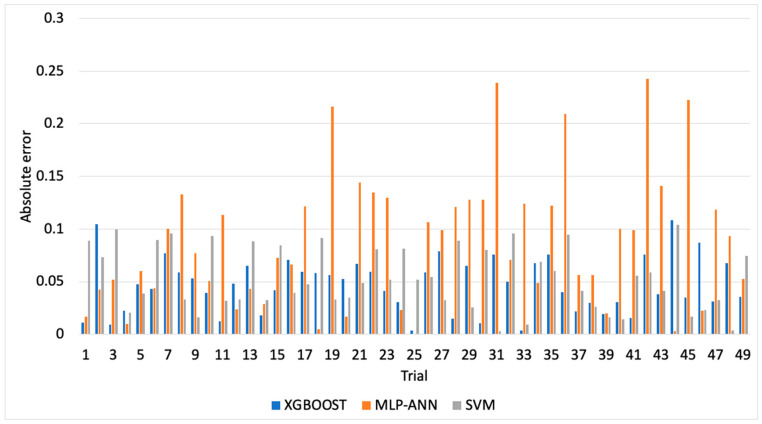
Comparison of errors in prediction of flank wear in for a copper workpiece.

**Figure 5:**
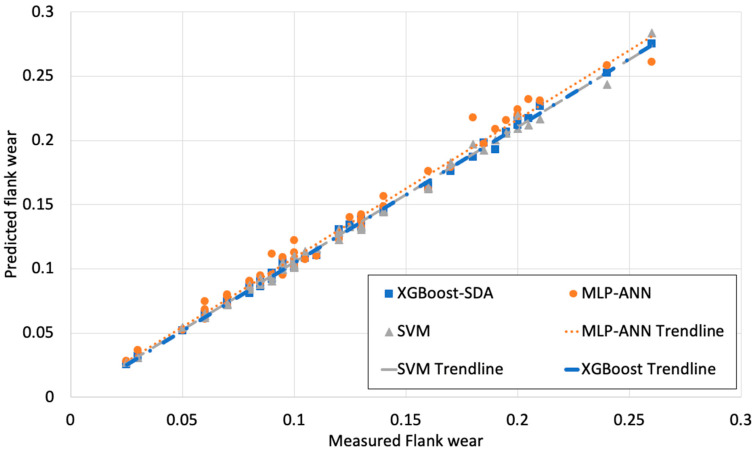
Measured Flank wear values vs. predicted Flank wear values of a drilling process of a copper workpiece.

**Figure 6:**
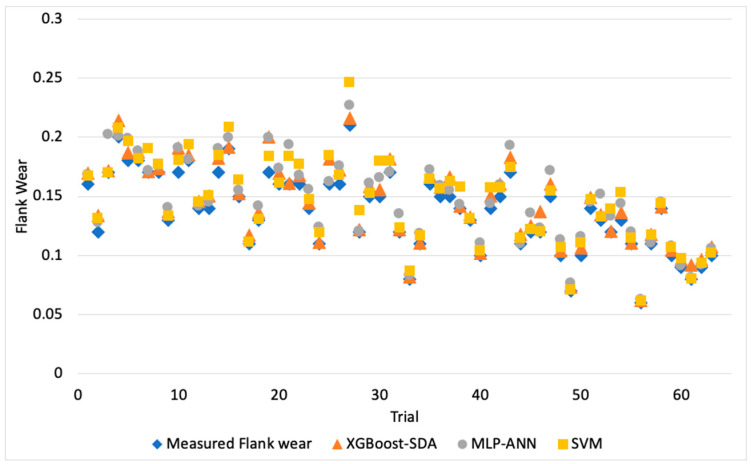
Flank wear values vs. predicted flank wear values of a drilling process (Cast Iron Workpiece).

**Figure 7:**
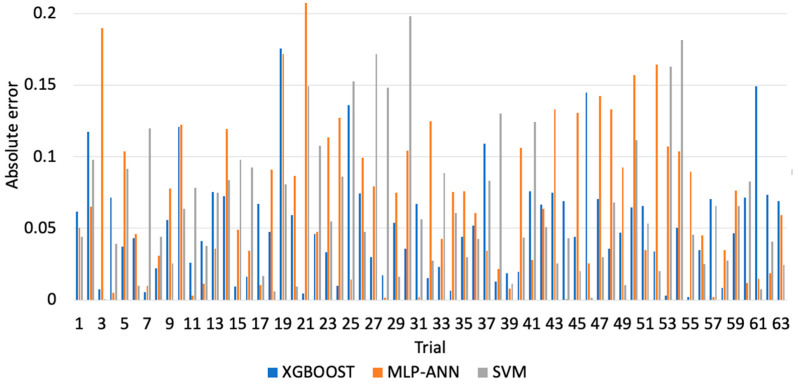
Comparison of errors in predicting flank wear of the cast iron workpiece.

**Figure 8:**
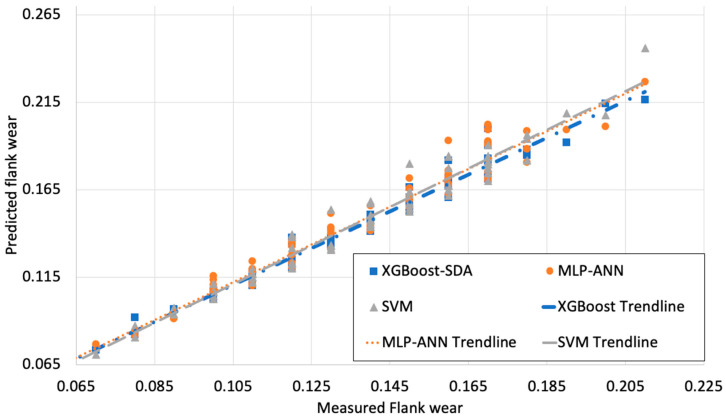
Measured Flank wear values vs. predicted flank wear values of a drilling process.

Figure 3, Figure 4 and Figure 5 illustrate the predicted flank wear values, the absolute error and the R^2^ plot of a copper workpiece. Similarly Figure 6, Figure 7 and Figure 8 present the predicted flank wear values, the absolute error and the R^2^ plot of a cast iron workpiece. 

Figure 3 and Figure 6 show the experimental flank wear values versus the predicted values that were observed. The predicted data points for the 49 trails for the copper workpiece and 63 trails for the cast iron agree with the experimental data, indicating the fitness of the model. They illustrate a comparison plot between the experimental and predicted flank wear values. Simulations were carried out to highlight the improvement of the proposed XGBoost-SDA over the SVM and MLP-ANN methods. Figure 4 presents a comparison of the errors in predicting flank wear for the XGBoost-SDA compared with the SVM and MLP-ANN for the copper workpiece. In predicting flank wear, the error with the XGBOOST-SDA model was the lowest (4.67%). It was found that the predictive XGBoost-SDA model was capable of better predictions of tool flank wear in the drilling process than the SVM and MLP-ANN models if they had been trained within the range. Additionally, it is noted that MLP-ANN resulted in higher prediction errors due to the fact that MLP-ANN requires tuning the number of layers as well as the number of neurons per layer.

Figure 5 and Figure 8 show the measured flank wear values versus the predicted flank wear values of a drilling process for the copper and cast-iron workpieces. The predicted and actual values were close to the absolute line for the XGBoost-SDA, SVM, and MLP-ANN models. The predicted flank wear obtained by XGBoost-SDA closely matches the actual values of measured flank wear, which visually endorses how well the XGBoost-SDA fits the validation dataset. It can be seen that the XGBoost-SDA, SVM, and MLP-ANN models could follow the tracks of actual tool wear effectively for both cases.

Figure 7 shows the comparison of errors in predicting flank wear for the XGBoost-SDA compared with the SVM and MLP-ANN for the cast iron workpiece. In predicting flank wear the error with the XGBOOST-SDA model was the lowest (5.25%). It was found that the predictive XGBoost-SDA model was capable of better predictions of tool flank wear in the drilling process than the SVM and MLP-ANN models if they had been trained within the range.

## 4. Conclusions

In this research we have presented a novel hybrid XGBoost-SDA prediction model for the flank wear of a drilling process. The strengths of XGBoost combined with the fast and accurate SDA showed superior performance in terms of the accurate prediction of flank wear for copper and cast-iron datasets. Simulations showed that XGBoost-SDA outperformed the state-of-the-art SVM and MLP-ANN models in terms of MAE, RMSE and the coefficient of determination R^2^. These characteristics are important for estimating flank wear with a given set of machine parameters and experimental trials. The predicted flank wear values were matched with the measured values in order to demonstrate the efficiency of the XGBoost-SDA. The predicted outcomes were found to be in close agreement with the experimental values. In the drilling of the copper workpiece, the MAE between experimental and predicted surface roughness values was 4.67%, while for the cast iron workpiece the MAE was 5.25%. A comparison of prediction accuracy between SVM, MLP-ANN, and the proposed XGBoots-SDA was carried out; it showed that the XGBoost-SDA resulted in greater accuracy in terms of the performance values in the drilling processes.

## Figures and Tables

**Table 1:** Nomenclature.

$R_{a}$	Arithmetic Surface Roughness (µm)
$R_{t}$	Maximum peak to valley height (µm)
V	Cutting Speed (m/min)
f	Feed Rate (mm/rev)
d	Depth of Cut (mm)
F	Thrust Force, N
M	Torque, Nm
$r_{2}$	Tool Nose Radius
α	major cutting edge angles
β	end cutting edge angle
MAE	Mean Root Square Error
RMSE	Root Mean Square Error
R^2^	Coefficient of Determination

**Table 2:** XGBoost hyperparameters.

Parameter	Description
*D*	maximum tree depth
γ	Regularization parameter to define the number of nodes in each tree
*K*	Number of trees
η	Learning rate
λ	Regularization parameter
*N*	Number of samples

**Table 3:** Spiral dynamic algorithm (SDA) optimization parameters.

Parameter	Description
θ	Rotation angle, 0≤θ≤2π
$k_{\max}$	Maximum iteration number.
*R*	Convergence rate of distance between a point and the origin, 0≤r≤1
$R_{i,j}$	Rotation matrix between *x_i_* and *x_j_* planes
M	Dimension of the search space

**Table 4:** Experimental data for Copper workpiece [17].

Trial	Spindle Speed (rpm)	Drill Diameter (mm)	Feed Rate (mm/rev)	Thrust Force (N)	Torque (Nm)	Measured Flank Wear (mm)
1	315	5	0.36	592	7.72	0.10
2	315	5	0.5	1210	13.39	0.08
3	315	5	0.71	1282	16.66	0.10
4	315	7.5	0.36	1866	12.74	0.16
5	315	7.5	0.5	1688	15.19	0.14
6	315	7.5	0.71	1828	17.15	0.12
7	315	10	0.36	3303	25.33	0.20
8	315	10	0.5	3413	29.54	0.21
9	315	10	0.71	3920	36.22	0.24
10	400	5	0.13	267	3.1	0.05
11	400	5	0.18	451	3.96	0.09
12	400	5	0.25	505	1.96	0.07
13	400	7.5	0.13	853	11.27	0.09
14	400	7.5	0.18	646	12.64	0.10
15	400	7.5	0.25	1051	16.54	0.10
16	400	10	0.13	2518	23.52	0.19
17	400	10	0.18	3921	26.78	0.20
18	400	10	0.25	4010	29.25	0.26
19	500	5	0.13	245	2.5	0.03
20	500	5	0.18	275	2.75	0.06
21	500	5	0.25	386	2.9	0.10
22	500	7.5	0.13	510	7.1	0.06
23	500	7.5	0.18	595	4.41	0.08
24	500	7.5	0.25	539	5.39	0.11
25	500	10	0.13	1925	19.253	0.11
26	500	10	0.18	3860	25.1	0.20
27	500	10	0.25	740	27.44	0.21
28	630	5	0.13	186	2.94	0.08
29	630	5	0.18	187	2.64	0.07
30	630	5	0.25	285	2.15	0.03
31	630	7.5	0.13	488	5.86	0.09
32	630	7.5	0.18	524	3.95	0.10
33	630	7.5	0.25	441	4.41	0.10
34	630	10	0.13	1258	10.11	0.12
35	630	10	0.18	1470	13.23	0.13
36	630	10	0.25	3077	5.68	0.18
37	800	5	0.5	1087	11.27	0.09
38	800	7.5	0.36	1666	8.66	0.13
39	800	7.5	0.5	1440	19.3	0.13
40	800	10	0.36	2234	22.34	0.16
41	800	10	0.5	2548	24.1	0.19
42	1000	5	0.36	421	4.21	0.06
43	1000	5	0.5	651	6.17	0.07
44	1000	7.5	0.36	554	5.39	0.10
45	1000	7.5	0.5	784	7.35	0.10
46	1000	7.5	0.71	970	8.05	0.12
47	1000	10	0.36	1460	12.25	0.14
48	1000	10	0.5	1960	18.13	0.13
49	1000	10	0.71	2009	20.58	0.17

**Table 5:** Performance comparison of XGBoost-SDA, support vector machines (SVM), and multilayer perceptron artificial neural networks (MLP-ANN).

Metric	XGBoost-SDA	SVM	MLP-ANN
MAE	4.67%	5.31%	8.87%
RMSE	5.32%	6.07%	10.86%
R^2^	0.9973	0.9952	0.9849

**Table 6:** Prediction results of the flank wear in the drilling process (Copper Workpiece).

Trial	Measured Flank Wear	XGBoost-SDA	MLP-ANN	SVM
1	0.10	0.10	0.10	0.11
2	0.08	0.09	0.08	0.09
3	0.10	0.10	0.11	0.11
4	0.16	0.16	0.16	0.16
5	0.14	0.15	0.15	0.15
6	0.12	0.13	0.13	0.13
7	0.20	0.22	0.22	0.22
8	0.21	0.22	0.23	0.21
9	0.24	0.25	0.26	0.24
10	0.05	0.05	0.05	0.06
11	0.09	0.09	0.10	0.09
12	0.07	0.07	0.07	0.07
13	0.09	0.09	0.09	0.09
14	0.10	0.10	0.10	0.10
15	0.10	0.10	0.11	0.11
16	0.19	0.20	0.20	0.19
17	0.20	0.21	0.22	0.21
18	0.26	0.28	0.26	0.28
19	0.03	0.03	0.04	0.03
20	0.06	0.06	0.06	0.06
21	0.10	0.10	0.11	0.10
22	0.06	0.06	0.07	0.07
23	0.08	0.08	0.09	0.08
24	0.11	0.11	0.11	0.11
25	0.11	0.11	0.11	0.12
26	0.20	0.21	0.22	0.21
27	0.21	0.23	0.23	0.22
28	0.08	0.08	0.09	0.09
29	0.07	0.08	0.08	0.07
30	0.03	0.03	0.03	0.03
31	0.09	0.10	0.11	0.09
32	0.10	0.11	0.11	0.11
33	0.10	0.10	0.11	0.10
34	0.12	0.13	0.13	0.13
35	0.13	0.13	0.14	0.13
36	0.18	0.19	0.22	0.20
37	0.09	0.09	0.10	0.09
38	0.13	0.13	0.14	0.13
39	0.13	0.13	0.13	0.13
40	0.16	0.17	0.18	0.16
41	0.19	0.19	0.21	0.20
42	0.06	0.07	0.08	0.06
43	0.07	0.07	0.08	0.07
44	0.10	0.11	0.10	0.11
45	0.10	0.10	0.12	0.10
46	0.12	0.13	0.12	0.12
47	0.14	0.14	0.16	0.15
48	0.13	0.14	0.14	0.13
49	0.17	0.18	0.18	0.18

**Table 7:** Experimental Data for Cast Iron workpiece [17].

Trial	Spindle Speed (rpm)	Drill Diameter (mm)	Feed Rate (mm/rev)	Thrust Force (N)	Torque (Nm)	Measured Flank Wear (mm)
1	250	9	0.13	1212.4	11.47	0.16
2	250	10	0.13	1677.3	16.01	0.12
3	250	11	0.13	1394.6	13.88	0.17
4	250	12	0.13	1578.2	15.62	0.2
5	250	9	0.18	1752.6	17.23	0.18
6	250	10	0.18	1869.7	18.64	0.18
7	250	11	0.18	2156.8	21.72	0.17
8	250	12	0.18	2163.4	21.41	0.17
9	250	9	0.25	2077.1	20.35	0.13
10	250	10	0.25	2824.2	28.11	0.17
11	250	11	0.25	2885.6	28.04	0.18
12	250	9	0.36	2816.7	28.22	0.14
13	250	10	0.36	3323.1	33.08	0.14
14	250	11	0.36	3001.4	29.14	0.17
15	250	12	0.36	3311.2	33.11	0.19
16	315	9	0.13	1185.2	11.43	0.15
17	315	10	0.13	1627.3	15.8	0.11
18	315	11	0.13	1342.9	13.61	0.13
19	315	12	0.13	1524.6	15.28	0.17
20	315	9	0.18	1707.8	16.97	0.16
21	315	10	0.18	1827.6	18.27	0.16
22	315	11	0.18	2097	21.03	0.16
23	315	12	0.18	2121.8	21.17	0.14
24	315	9	0.25	2025.8	20.06	0.11
25	315	10	0.25	2786.7	27.84	0.16
26	315	11	0.25	2753.8	27.68	0.16
27	315	12	0.25	2612.6	26.21	0.21
28	315	9	0.36	2778	27.82	0.12
29	315	10	0.36	3284.2	32.95	0.15
30	315	11	0.36	2860.1	28.55	0.15
31	315	12	0.36	3270	32.99	0.17
32	400	9	0.13	1150.9	11.22	0.12
33	400	10	0.13	1215.6	11.18	0.08
34	400	11	0.13	1318.6	13.04	0.11
35	400	12	0.13	1464.3	14.39	0.16
36	400	9	0.18	1486.4	15.01	0.15
37	400	10	0.18	1547.7	15.71	0.15
38	400	11	0.18	2067	20.71	0.14
39	400	12	0.18	2114.6	18.64	0.13
40	400	9	0.25	1642.8	16.36	0.1
41	400	10	0.25	1715.2	17.08	0.14
42	400	11	0.25	2538.9	25.42	0.15
43	400	12	0.25	2558.6	23.58	0.17
44	400	9	0.36	1721.3	17.64	0.11
45	400	10	0.36	1782.6	17.95	0.12
46	400	11	0.36	2752.7	27.66	0.12
47	400	12	0.36	2924.3	24.92	0.15
48	500	9	0.13	1088.1	10.67	0.1
49	500	10	0.13	1188.3	11.06	0.07
50	500	11	0.13	1254.9	12.54	0.1
51	500	12	0.13	1277.8	13.28	0.14
52	500	9	0.18	1435.1	14.66	0.13
53	500	10	0.18	1504.8	15.11	0.12
54	500	11	0.18	1556.8	18.32	0.13
55	500	12	0.18	1624.3	18.51	0.11
56	500	9	0.25	1588.3	16.04	0.06
57	500	10	0.25	1668.9	16.85	0.11
58	500	11	0.25	1724.3	23.41	0.14
59	500	12	0.25	1856.3	23.51	0.1
60	500	9	0.36	1669.8	17.12	0.09
61	500	10	0.36	1754.8	17.69	0.08
62	500	11	0.36	1869.4	24.65	0.09
63	500	12	0.36	2005.4	24.78	0.1

**Table 8:** Performance comparison of XGBoost-SDA, SVM, and MLP-ANN.

Metric	XGBoost-SDA	SVM	MLP-ANN
MAE	5.25%	6.51%	7.13%
RMSE	6.49%	8.07%	8.83%
R^2^	0.9756	0.9633	0.9553

**Table 9:** Prediction results of the flank wear in drilling process (Cast Iron Workpiece).

Trial	Measured Flank Wear	XGBoost-SDA	MLP-ANN	SVM
1	0.16	0.17	0.17	0.17
2	0.12	0.13	0.13	0.13
3	0.17	0.17	0.20	0.17
4	0.2	0.21	0.20	0.21
5	0.18	0.19	0.20	0.20
6	0.18	0.19	0.19	0.18
7	0.17	0.17	0.17	0.19
8	0.17	0.17	0.18	0.18
9	0.13	0.14	0.14	0.13
10	0.17	0.19	0.19	0.18
11	0.18	0.18	0.18	0.19
12	0.14	0.15	0.14	0.15
13	0.14	0.15	0.14	0.15
14	0.17	0.18	0.19	0.18
15	0.19	0.19	0.20	0.21
16	0.15	0.15	0.16	0.16
17	0.11	0.12	0.11	0.11
18	0.13	0.14	0.14	0.13
19	0.17	0.20	0.20	0.18
20	0.16	0.17	0.17	0.16
21	0.16	0.16	0.19	0.18
22	0.16	0.17	0.17	0.18
23	0.14	0.14	0.16	0.15
24	0.11	0.11	0.12	0.12
25	0.16	0.18	0.16	0.18
26	0.16	0.17	0.18	0.17
27	0.21	0.22	0.23	0.25
28	0.12	0.12	0.12	0.14
29	0.15	0.16	0.16	0.15
30	0.15	0.16	0.17	0.18
31	0.17	0.18	0.17	0.18
32	0.12	0.12	0.13	0.12
33	0.08	0.08	0.08	0.09
34	0.11	0.11	0.12	0.12
35	0.16	0.17	0.17	0.16
36	0.15	0.16	0.16	0.16
37	0.15	0.17	0.16	0.16
38	0.14	0.14	0.14	0.16
39	0.13	0.13	0.13	0.13
40	0.1	0.10	0.11	0.10
41	0.14	0.15	0.14	0.16
42	0.15	0.16	0.16	0.16
43	0.17	0.18	0.19	0.17
44	0.11	0.12	0.11	0.11
45	0.12	0.13	0.14	0.12
46	0.12	0.14	0.12	0.12
47	0.15	0.16	0.17	0.15
48	0.1	0.10	0.11	0.11
49	0.07	0.07	0.08	0.07
50	0.1	0.11	0.12	0.11
51	0.14	0.15	0.14	0.15
52	0.13	0.13	0.15	0.13
53	0.12	0.12	0.13	0.14
54	0.13	0.14	0.14	0.15
55	0.11	0.11	0.12	0.12
56	0.06	0.06	0.06	0.06
57	0.11	0.12	0.11	0.12
58	0.14	0.14	0.14	0.14
59	0.1	0.10	0.11	0.11
60	0.09	0.10	0.09	0.10
61	0.08	0.09	0.08	0.08
62	0.09	0.10	0.09	0.09
63	0.1	0.11	0.11	0.10
